# Development and Testing of a Compact Autorefractor Based on Double-Pass Imaging

**DOI:** 10.3390/s23010362

**Published:** 2022-12-29

**Authors:** Linus Emmerich, Arne Ohlendorf, Alexander Leube, Nikolai Suchkov, Siegfried Wahl

**Affiliations:** 1 Institute for Ophthalmic Research, Eberhard Karls University Tuebingen, Elfriede-Aulhorn-Str. 7, 72076 Tuebingen, Germany; 2 Carl Zeiss Vision International GmbH, Turnstr. 27, 73430 Aalen, Germany

**Keywords:** optometry, autorefraction, double-pass imaging, point spread function, modulation transfer function

## Abstract

Autorefraction is an objective way to determine the refractive error of the eye, without the need for feedback by the patient or a well-educated practitioner. To make refractive measurements more accessible in the background of the growing prevalence of myopia, a compact autorefractor was built, containing only few optical components and relying on double-pass imaging and the physical properties of the point-spread function and digital image processing instead. A method was developed to analyze spherical defocus as well as the defocus and angle of astigmatism. The device was tested using calibrator eye models in a range of ± 15 D spherical defocus and −3 D astigmatic defocus. Reliable results could be achieved across the whole measurement range, with only a small increase in deviation toward high values of refractive errors, showing the feasibility of a PSF-based approach for a compact and low-cost solution for objective measurements of refractive error.

## 1. Introduction

Refractive error of the human eye is a problem that is affecting people of all ages across the world. Studies have shown that uncorrected refractive error is the second most common cause for blindness, and the most common cause for mild and severe vision impairment worldwide [1,2]. When not treated properly, negative effects on ocular health and visual acuity can be the consequence [3]. Additionally, uncorrected refractive error also has economic influence, as it impedes the daily routines and the workflows of people affected. In that way, it can lower the productivity within a society [4,5].

The prevalence of myopia is rising [6], and it is expected that 50% of the world population will be myopic by 2050 [7]. Especially in Asia, a steep rise of the prevalence of myopia has been reported together with a rising number of cases of high myopia [8,9]. Regarding the total numbers of people suffering from uncorrected refractive errors and the associated individual and economic consequences, there is a clear need to measure refractive error and reach as many people as possible even in remote locations that still only provide limited healthcare.

Refractive errors can be measured either subjectively, relying on the feedback of the subject, or objectively. In that latter case, no subjective feedback is needed and the measurement results will either be determined by the practitioner or generated electronically by the device itself. When working with objective methods, results will mainly depend on the device used, while subjective methods depend strongly on the skills and behavior of the examiner as well as the cooperation of the subject. When trying to expand the reach of measurements of refractive error, several factors need to be taken into account. Subjective methods of refraction require the subject to provide a sensible feedback to the procedure. Additionally, a skilled practitioner is needed to perform the measurement. Those are serious limitations in some less developed regions of the world, which is why objective methods of measurement should be prioritized. Although only taking into account the optical components of the visual system without being able to integrate the neural component, it has been shown that there is good agreement between objective and subjective methods of refractive measurements [10,11]. Even if subjective refraction is still regarded as the gold standard today [12], the scope of reaching people in remote regions that do not have access to good healthcare yet clearly points towards an objective approach. Retinoscopy is a quick method to determine refractive error without relying on the subject’s feedback or requiring large and heavy instruments. On the downside, it can only be performed by an educated practitioner. Many modern devices to objectively assess the refractive errors of the eye nowadays work with a wavefront sensor [13]. They use a microlens array to focus an incoming beam of light onto multiple spots on a sensor behind the array. The displacement of each spot from its predetermined position can then be determined and utilized to calculate the defocus of the incoming beam of light. Wavefront sensors are very expensive, and a compromise needs to be made between the accuracy and the measurement range. By choosing a large size of lenses in the array, a high measurement range can be achieved at the cost of reduced accuracy. Choosing an array of very small lenses, a high accuracy can be achieved with a limited range of measurable defocus. Another downside of wavefront sensors is the large size. Apart from some portable models [14,15,16], most of these systems are table-based and are designed to be set up in clinics or a doctor’s examination room. They offer little portability, and accordingly, they are not suitable to be deployed in regions with poor infrastructure for healthcare. For those circumstances, a “smart” solution for objective refraction is required, providing portability, simple operation and accessibility, i.e., low-cost components. Different handheld devices exist already, e.g., SVOne [17], a smartphone-based wavefront abberrometer, or Eye Netra [18]. While also smartphone-based, Eye Netra requires the user to subjectively align a pattern on the screen, making it a subjective form of measurement. As a result, the device will print out spherical and astigmatic defocus as well as the angle of astigmatism. Double-pass systems for refractive measurements have already been explored decades ago [19,20,21], and while there are stationary devices that work by analyzing the Point Spread Function of light that is scattered from the retina [22,23], to our knowledge, a portable and affordable solution is not commercially available yet. The presented research introduces a compact, portable and low-cost device to measure objective refraction, relying on double-pass imaging. The new analytical approach is designed to deliver quick and reliable measurements of spherical and astigmatic defocus, as well as principal meridians, without the need for user feedback or a skilled examiner.

## 2. Materials and Methods

### 2.1. Physical Concept

As the point spread function (PSF) is a measure of the quality of any optical system, it contains information about the properties of the complete optical system it originates from. The described solution for an objective autorefractor uses this approach to analyze the PSF that is generated on a person’s retina with a laser beam in order to gain information about the refractive error of the eye. This concept is used in combination with a focus adjustable lens that is used to compensate for the defocus of the examined eye. The focus of the adjustable lens is changed with the aim of minimizing the resulting defocus originating from the adjustable lens itself and the refractive error of the measured eye. When the overall defocus in the system is smallest, there will be a peak in intensity of the observed PSF. In this condition, the focal power of the adjustable lens equals the inverse power of the spherical refractive error of the eye. In the case of astigmatism, two peak intensities of the PSF can be observed at different values of defocus and at angles separated by 90°. By calculating the modulation transfer function (MTF) from the PSF, a metric can be evaluated that correlates well with functional visual quality and provides information about angle-dependent retinal image quality [24].

### 2.2. Setup

**Figure 1:**
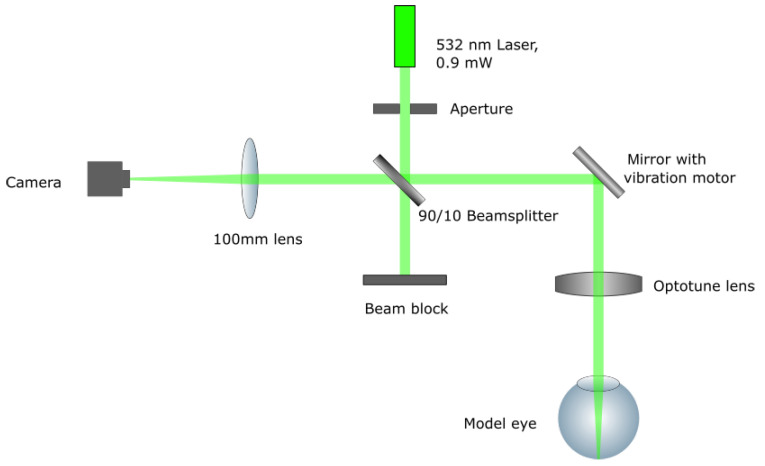
Setup schematic.

Figure 1 represents the path of light in the PSFRx system. Following the path of the light, the system begins at the laser source, a 532 nm laser diode with an output of 0.9 mW. An adjustable aperture is used to create a circular beam shape with a diameter of 2 mm. Behind the aperture, a beamsplitter with a transmission rate of 90% redirects the beam toward the eye. From the beamsplitter, the light is sent to a mirror, which is coupled to a vibration motor (Shenzhen Xinhailong CO., LTD., Shenzhen, China). That way, speckle can be reduced effectively [25], without the need for more expensive components such as variable focus lenses [26]. The Optotune EL-3 (Optotune Switzerland AG, Dietikon, Switzerland) [27] as a focus-adjustable element is located right in front of the eye. After the incoming beam is scattered back at the retina of the examined eye, it passes the Optotune lens, the vibrating mirror and the beamsplitter again. Finally, the beam is focused onto a camera sensor with a 100 mm lens. Both the focus-adjustable lens and the camera are connected to the same computer to be controlled simultaneously. The camera model DMK 27AUP031 from The Imaging Source (The Imaging Source Europe GmbH, Bremen, Germany) is used with a resolution of 1920 × 1080 pixels. It is equipped with a CMOS sensor with a pixel size of 2.2 μm × 2.2 μm. For validation, objective calibrator eye models with different values of spherical and astigmatic defocus (National institute of Metrology, Beijing, China) were used. These eye models are built especially for the calibration of refractometers and meet the requirements of the European Standard for eye refractometers (ISO 10342:2010 Ophthalmic instruments—Eye refractometers) [28]. Therefore, they offer very high precision in optical power. Due to the optical quality and the possibility of steady fixation in the system, they are ideal tools for the evaluation of this setup.

### 2.3. Software

For fast and repeatable measurements, a code was created in C# (Microsoft Corporation, Redmond, WA, USA) using the provided source code from Optotune for the lens and source code from The Imaging-Source for the camera. The exposure time of the camera and the timeout between changing the current of the lens and taking an image are determined in the code, while the interface allows the user to set custom values for the starting current, step size and end current of the lens, as well as the direction and number of repetitions of the measurement.

To gain information not only about spherical defocus, but also about the astigmatic power as well as the orientation of the two principal meridians, it is necessary to analyze the intensity of the captured PSFs for different angles individually. The analysis of the captured images is performed with MatLab (Version R2019b, The MathWorks, Inc., Natick, MA, USA). As a first step, all images taken in the measurement are imported. For better accuracy, the images from ten repetitions can be imported to calculate their average for further processing. After that, all images are cropped from 1920 × 1080 pixels to 1080 × 1080 pixels and are padded with a black frame of 1080 pixels on all sides. From these images, the Modulation Transfer function is calculated. The MatLab function *fft2* is used to calculate the discrete two-dimensional Fourier transform of the PSF image. The results are transformed into absolute values before normalizing the resulting matrix. In each resulting MTF image, the pixel values of the horizontal center line of pixels are summed up. This value is saved, and the image is rotated by one degree counterclockwise before the center line of pixels is added up again. This is repeated until reaching a total rotation of 180°. From all pixel values, a matrix is created with the dimensions of the number of images × 180. The values at 90° are replaced by the average of the values at 89° and 91°, and the values at 180° are replaced by the average of the values at 179° and 1° to avoid Artifacts resulting from the Fourier-transform at these angles. As the function *fftshift* rearranges the fourier transform shifting the zero-frequency component to the center of the array, artifacts occur at 90° and 180° where the former edges of the MTF image meet. The matrix is then expanded, using the same pixel values again for angles between 180° and 360°. This way, angles around 0°/180° can be determined more accurately. Finally, a Gaussian filter is applied to smooth the matrix.

**Figure 2:**
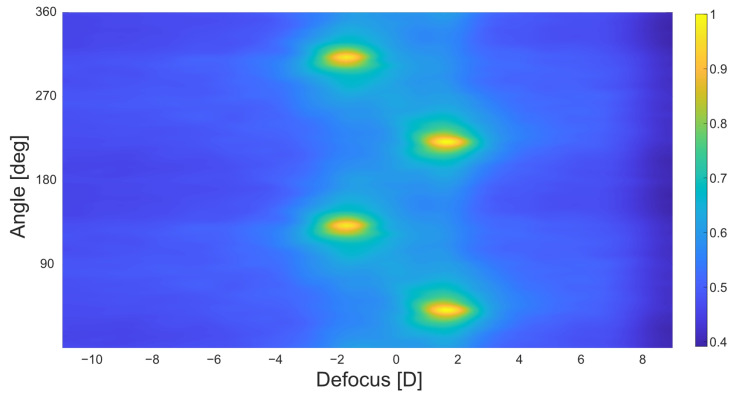
Example for the intensity matrix of an astigmatic lens.

To find the location of the maximum values, the variance of the gradient of the matrix is calculated. The location of the highest variance resembles the location of the peaks. After finding the location of the total maximum in the matrix, a second peak is located in the area between 80° and 100° shifted from the first one to consider the second principal meridian for an astigmatic defocus. As the focus-adjustable lens can only be controlled by adjusting the current, at the final stage of the process, the current at the location of peaks needs to be converted into values of optical power. Spherical defocus of the focus-adjustable lens is calculated for the image numbers of the peaks in order to print out defocus and angles as a result. Last, the peak with the higher value of defocus is interpreted as the value of spherical defocus. The difference in optical power between both meridians is interpreted as the value of astigmatic defocus and the angle of the peak with lower value is interpreted as the principal meridian of astigmatism, as shown in Figure 2.

## 3. Results

The system was validated following the European Standard for eye refractometers (ISO 10342:2010 Ophthalmic instruments—Eye refractometers) [28]. To fulfill the standards requirements of covering a range of ± 15 D spherical defocus and −3 D of cylinder, measurements were performed with a set of calibrator eye models (National institute of Metrology, China) with spherical defocus values of 0D, ± 2.5D, ±5 D, ±10 D and ±15 D, as well as −3 D of astigmatic defocus at 0° and 90°. A mount for the eye models is fixed in the system 15 mm behind the Optotune lens. That way, the models can be changed quickly without the need for individual position adjustments after the initial calibration. The models are automatically centered correctly and keep exactly the same distance to the focus adjustable lens.

**Figure 3:**
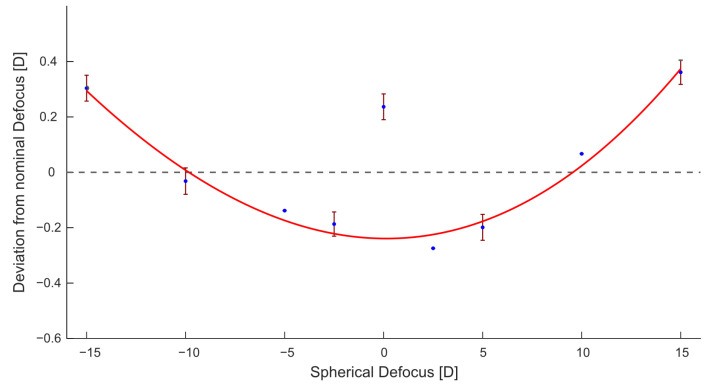
Results of all spherical defocus measurements.

For each eye model, the current at the adjustable lens when compensating for the introduced defocus is checked roughly with the live image of the camera sensor. That way, the required measurement range is decreased significantly, thus speeding up both the measurement process and the analysis. The results represented in Table 1 were achieved by averaging 10 consecutive measurements of each eye model; Figure 3 shows the distribution of all measurements for eye models with spherical defocus.

For all measurements, a settling time of 50 ms is set between changing the current at the lens and taking a picture with the camera. The power of the lens is adjusted in increments of 0.5 mA, which translates to approximately 0.1 D. One single measurement run across a range of 5 D takes 5 s that way.

## 4. Discussion

In the European Standard for eye refractometers (ISO 10342:2010 Ophthalmic instruments—Eye refractometers) [28], the maximum limit of deviation from the nominal defocus of the eye model is given as 0.25 D for all values of defocus except ±15 D, which allows for a deviation of 0.5 D. These regulations are met in all cases besides the measurement of the 2.5 D eye model, which is marked red in Table 1. The standard deviations between the 10 single runs of each measurement lie between 0 D and 0.083 D with a single outlier at 0.16 D standard deviations. As the deviations from the nominal lens values are both positive and negative, there seems to be no systematic error in the system and the tolerances in the measurement results appear to be associated with inaccuracies in the setup alignment. Furthermore, the current applied to the Optotune lens seems to result in slightly different values of focal power, depending on the current that was applied before and the range that is covered within a measurement run. Recent research on the characterization of Optotune’s electrically focus-tunable lenses supports this observation by confirming hysteresis in three different lens models [29,30]. When treating the measurement of 0 D as an outlier, Figure 3 shows an obvious trend for the lens measurements to increase toward higher absolute values of defocus compared to the nominal values of the model eyes. As the initial lens calibration was performed across the whole measurement range, deviations appear both negative in the center region as well as positive in the distal regions of the plot. Considering the construction of the lens, it is likely that the optical power is more accurate for lower values of defocus, while there may be nonlinear factors such as the elasticity of the flexible membrane of the lens resulting in a higher divergence of optical power toward higher values of defocus.

As the measurements in this study were performed under ideal conditions with high quality eye models, a decrease in accuracy and repeatability of the PSFRx can be expected when working with human subjects. Accommodation as well as eye movements and blinking of the subjects are factors that need to be taken into account. Additionally, the fixation of the subject’s head will be more difficult than fixating a model eye in front of the system. While the problem of blinking can be settled by taking multiple measurements and eliminating the faulty runs, other challenges can be more difficult to control. Steady positioning and correct centering of the measured eyes as well as an efficient accommodation control will be crucial requirements to achieve good results. Still, a mismatch between the results of bench based and real life qualifications can be expected. Another point to consider is the increased challenge to evaluate measurement accuracy in a clinical setup. Without model eyes with a precisely defined value of defocus, a statement about the quality of a system for objective measurements of refraction can only be made by comparing measurement results to those of other devices or to subjective refraction. Variations can be observed when investigating inter-device agreement between different devices or methods of refraction, especially in regard to the limits of agreement between different devices of the same technology or when comparing devices of different technology [31,32,33]. Therefore, the results presented in this study need to be complemented by further investigations about the performance in the field to be able to make a comprehensive statement about the real life quality of PSFRx refraction.

## 5. Conclusions

With the system described, the concept of PSF-based refraction was successfully executed. Key elements are the focus-adjustable lens, as well as the vibration motor, for speckle reduction. In comparison to current state-of-the-art systems, this novel concept promises a very compact and low-cost solution. For the prototype, only commercial off-the-shelf components are used with a total cost of less than 1000$. In addition, there is virtually no limit for the defocus that can be measured, as the focus-adjustable lens can be paired with an additional fixed focal lens to shift the range into any desired direction. Finally, unlike devices using a Hartmann–Shack sensor, the measurement accuracy does not depend on the measurement range, and it also does not decrease significantly for higher values of defocus. Current limitations of accuracy are only technical and can be overcome by applying precision-built components and refining alignment. On top, the setup can be easily upgraded for subjective measurements of refraction.

To be able to perform measurements on human subjects, some changes need to be made in the system. Foremost, the laser source needs to be changed from 532 nm to an infrared source. This will reduce the energy that is put into the eye and, at the same time, avoid accommodation to the defocus of the laser beam. To deal with the problem of accommodation more effectively, a fixation target should be included. The system is already prepared to be extended in that regard, as the vibrating mirror can be replaced by a short-pass mirror. This allows for the placement of a fixation target behind it, while reflecting the infrared laser beam toward the eye and the camera, respectively.

To better comprehend the accuracy of the focus-adjustable lens toward higher values of defocus, a separate set of measurements would be beneficial, collecting more data points with smaller intervals in optical power.

## Figures and Tables

**Table 1:** Measurement results with test eyes.

Nominal [D]			Measured Average [D]			Deviation [D]	
**Sph**	**Cyl**	**Angle [deg]**	**Sph**	**Cyl**	**Angle [deg]**	**Sph**	**Cyl**
−15			−15.304	−0.115		0.304	0.115
−10			−9.968	−0.048		−0.032	0.048
−5			−4.862	0.000		−0.138	0.000
−2.5			−2.313	−0.067		−0.187	0.067
0			−0.237	−0.038		0.237	0.038
2.5			2.774	0.000		−0.274	0.000
5			5.199	−0.038		−0.199	0.038
10			9.933	−0.038		0.067	0.038
15			14.639	−0.067		0.361	0.067
	−3	0	−0.070	−3.217	0.100	0.070	0.217
	−3	90	−0.032	−3.207	91.000	0.032	0.207

## Data Availability

Data underlying the results presented in this paper are not publicly available at this time, but may be obtained from the authors upon reasonable request.

## References

[1] Bourne R.R., Stevens G.A., White R.A., Smith J.L., Flaxman S.R., Price H., Jonas J.B., Keeffe J., Leasher J., Naidoo K. (2013). Causes of vision loss worldwide, 1990–2010: A systematic analysis. Lancet Glob. Health.

[2] Resnikoff S., Pascolini D., Mariotti S.P., Pokharel G.P. (2008). Global magnitude of visual impairment caused by uncorrected refractive errors in 2004. Bull. World Health Organ..

[3] Wong T.Y., Ferreira A., Hughes R., Carter G., Mitchell P. (2014). Epidemiology and disease burden of pathologic myopia and myopic choroidal neovascularization: An evidence-based systematic review. Am. J. Ophthalmol..

[4] Smith T., Frick K., Holden B., Fricke T., Naidoo K. (2009). Potential lost productivity resulting from the global burden of uncorrected refractive error. Bull. World Health Organ..

[5] Fricke T., Holden B., Wilson D., Schlenther G., Naidoo K., Resnikoff S., Frick K. (2012). Global cost of correcting vision impairment from uncorrected refractive error. Bull. World Health Organ..

[6] Vitale S., Ellwein L., Cotch M.F., Ferris F.L., Sperduto R. (2008). Prevalence of refractive error in the United States, 1999–2004. Arch. Ophthalmol..

[7] Holden B.A., Fricke T.R., Wilson D.A., Jong M., Naidoo K.S., Sankaridurg P., Wong T.Y., Naduvilath T.J., Resnikoff S. (2016). Global prevalence of myopia and high myopia and temporal trends from 2000 through 2050. Ophthalmology.

[8] Pan C.W., Dirani M., Cheng C.Y., Wong T.Y., Saw S.M. (2015). The age-specific prevalence of myopia in Asia: A meta-analysis. Optom. Vis. Sci..

[9] Hung T. (2001). Epidemiologic study of the prevalence and severity of myopia among schoolchildren in Taiwan in 2000. J. Formos. Med. Assoc..

[10] Cleary G., Spalton D., Patel P., Lin P.F., Marshall J. (2009). Diagnostic accuracy and variability of autorefraction by the Tracey Visual Function Analyzer and the Shin-Nippon NVision-K 5001 in relation to subjective refraction. Ophthalmic Physiol. Opt..

[11] Ohlendorf A., Leube A., Wahl S. (2016). Steps towards smarter solutions in optometry and ophthalmology—Inter-device agreement of subjective methods to assess the refractive errors of the eye. Healthcare.

[12] Rodriguez-Lopez V., Dorronsoro C. (2022). Beyond traditional subjective refraction. Curr. Opin. Ophthalmol..

[13] Artal P. (2017). Handbook of Visual Optics: Instrumentation and Vision Correction.

[14] Schimitzek T., Wesemann W. (2002). Clinical evaluation of refraction using a handheld wavefront autorefractor in young and adult patients. J. Cataract. Refract. Surg..

[15] Durr N.J., Dave S.R., Vera-Diaz F.A., Lim D., Dorronsoro C., Marcos S., Thorn F., Lage E. (2015). Design and clinical evaluation of a handheld wavefront autorefractor. Optom. Vis. Sci..

[16] Rubio M., Hernández C.S., Seco E., Perez-Merino P., Casares I., Dave S.R., Lim D., Durr N.J., Lage E. (2019). Validation of an affordable handheld wavefront autorefractor. Optom. Vis. Sci..

[17] Ciuffreda K.J., Rosenfield M. (2015). Evaluation of the SVOne: A handheld, smartphone-based autorefractor. Optom. Vis. Sci..

[18] Bastawrous A., Leak C., Howard F., Kumar V. (2012). Validation of near eye tool for refractive assessment (NETRA)–pilot study. J. Mob. Technol. Med..

[19] McBRIEN N.A., Millodot M. (1985). Clinical evaluation of the Canon Autoref R-1. Am. J. Optom. Physiol. Opt..

[20] Santamaría J., Artal P., Bescós J. (1987). Determination of the point-spread function of human eyes using a hybrid optical–digital method. JOSA A.

[21] Williams D.R., Brainard D.H., McMahon M.J., Navarro R. (1994). Double-pass and interferometric measures of the optical quality of the eye. JOSA A.

[22] Wolffsohn J., Hunt O., Gilmartin B. (2002). Continuous measurement of accommodation in human factor applications. Ophthalmic Physiol. Opt..

[23] Visiometrics HD Analyzer. https://www.ophthalmologyweb.com/1315-News/120477-Visiometrics-launches-the-HD-Analyzer-A-next-generation-Optical-Quality-Analysis-System/.

[24] Artal P. (2017). Handbook of Visual Optics: Fundamentals and Eye Optics.

[25] Halpaap D., García-Guerra C.E., Vilaseca M., Masoller C. (2019). Speckle reduction in double-pass retinal images. Sci. Rep..

[26] García-Guerra C.E., Aldaba M., Arjona M., Pujol J. (2015). Speckle reduction in double-pass retinal images using variable-focus lenses. J. Eur. Opt. Soc.-Rapid Publ..

[27] Optotune Switzerland AG Optotune EL-3-10. https://www.optotune.com/el-3-10-lens.

[28] (2010). Ophthalmic Instruments—Eye Refractometers.

[29] Marín J.M., Torres-Sepúlveda W., Henao J., Mira-Agudelo A., Rueda E. (2020). Optical characterization of electro-optics lenses for researching in optics. Optical System Alignment, Tolerancing, and Verification XIII.

[30] Torres-Sepúlveda W.A., Escobar J.D.H., Marín J.A.M., Mira-Agudelo A., Muñoz E.A.R. (2020). Hysteresis characterization of an electrically focus-tunable lens. Opt. Eng..

[31] Jeganathan S.E., Valikodath N., Niziol L.M., Hansen V.S., Apostolou H., Woodward M.A. (2018). Accuracy of a Smartphone-Based Autorefractor Compared to Gold-Standard Refraction. Optom. Vis. Sci. Off. Publ. Am. Acad. Optom..

[32] Kumar R.S., Moe C.A., Kumar D., Rackenchath M.V., AV S.D., Nagaraj S., Wittberg D.M., Stamper R.L., Keenan J.D. (2021). Accuracy of autorefraction in an adult Indian population. PLoS ONE.

[33] Gwiazda J., Weber C. (2004). Comparison of spherical equivalent refraction and astigmatism measured with three different models of autorefractors. Optom. Vis. Sci..

